# Cystic fibrosis physicians’ perspectives on the timing of referral for lung transplant evaluation: a survey of physicians in the United States

**DOI:** 10.1186/s12890-017-0367-9

**Published:** 2017-01-19

**Authors:** Kathleen J. Ramos, Ranjani Somayaji, Erika D. Lease, Christopher H. Goss, Moira L. Aitken

**Affiliations:** 1 Department of Medicine, Division of Pulmonary and Critical Care Medicine, University of Washington Medical Center, 1959 NE Pacific Street, Box 356522, Seattle, WA 98195 USA; 2 Department of Medicine, University of Calgary, 3330 Hospital Drive, Calgary, AB Canada; 3 Seattle Children’s Research Institute, 2001 Eighth Avenue, Suite 400, Seattle, WA 98121 USA; 4 Department of Pediatrics, University of Washington, 6200 NE 74th St, Ste 110, Seattle, WA 98115 USA

**Keywords:** Cystic fibrosis, Lung transplantation, Referral, Physician survey

## Abstract

**Background:**

Prior studies reveal that a significant proportion of patients with cystic fibrosis (CF) and advanced lung disease are not referred for lung transplant (LTx) evaluation. We sought to assess expert CF physician perspectives on the timing of LTx referral and investigate their LTx knowledge.

**Methods:**

We developed an online anonymous survey that was distributed by the Cystic Fibrosis Foundation (CFF) to the medical directors of all CFF-accredited care centers in the United States in 2015. The survey addressed only adult patients (≥18 years old) and was sent to 119 adult CF physicians, 86 CFF-affiliated CF physicians (who see adults and children, but have smaller program sizes than adult or pediatric centers), and 127 pediatric CF physicians (who see some adults, but mostly children). The focus of the questions was on CFF-care center characteristics, physician experience and indications/contraindications to referral for LTx evaluation.

**Results:**

There were 114/332 (34%) total responses to the survey. The response rates were: 57/119 (48%) adult physicians, 12/86 (14%) affiliate physicians and 43/127 (34%) pediatric physicians; 2 physicians did not include their CFF center type. Despite the poor ability of FEV_1_ < 30% to predict death within 2 years, 94% of responding CF physicians said they would refer an adult patient for LTx evaluation if the patient’s lung function fell to FEV_1_ < 30% predicted. Only 54% of respondents report that pulmonary hypertension would trigger referral. Pulmonary hypertension is an internationally recommended indication to list a patient for LTx (not just for referral for evaluation). Very few physicians (*N* = 17, 15%) employed components of the lung allocation score (LAS) to determine the timing of referral for LTx evaluation. Interestingly, patient preference not to undergo LTx was “often” or “always” the primary patient-related reason to defer referral for LTx evaluation for 41% (47/114) of respondents.

**Conclusions:**

Some potential barriers to timely LTx referral for patients with CF include physician knowledge regarding non-lung function-based recommendations related to timing of referral and listing for LTx, and patient preference not to undergo LTx. Further exploration of physician-level and CF patient-level barriers to timely LTx referral is warranted.

**Electronic supplementary material:**

The online version of this article (doi:10.1186/s12890-017-0367-9) contains supplementary material, which is available to authorized users.

## Background

Cystic fibrosis (CF) is a progressive, lethal genetic disease that affects an estimated one in 3500 newborns in the United States (US) [1]. Approximately 81–84% of patients diagnosed with CF are treated at Cystic Fibrosis Foundation (CFF)-accredited Programs nationwide, where expert care and specialized disease management is evidence-based via care guidelines [1, 2]. Physicians who work at CFF-accredited centers are internists or pediatricians often with subspecialty pulmonary medicine training. The CFF has a mission “to find a cure for CF and improve the quality of life for people with the disease” [1].

Despite therapeutic advances and increasing life expectancy, respiratory failure remains the most frequent cause of premature death among patients with CF [1, 3]. Lung transplantation (LTx) is a treatment option for select patients with CF and advanced lung disease. The decision to pursue organ transplantation is a complex balance of patient preference and physician judgment of medical indication and patient candidacy. However, we have yet to fully understand the factors leading to referral for lung transplantation. Notably, a study of patients in the CFF Patient Registry demonstrated that only 65% of patients who decidedly met medical criteria (forced expiratory volume in one second (FEV_1_) <30% predicted for two consecutive years) were referred for LTx evaluation [4]. Among patients with FEV_1_ < 30% predicted, more US CF patients die without LTx each year after reaching this threshold than undergo transplantation [5]. Disparities in access to LTx referral based on socioeconomic status have been highlighted, but it remains unclear whether physician or patient factors led to non-referral of these patients with CF and advanced lung disease [4]. A retrospective analysis of 256 deaths among patients with CF in France, 2007–2010, revealed that half of the deaths (*n* = 129) occurred in patients who did not undergo LTx [6]. Among the patients who died without listing for LTx despite having an indication for transplant, LTx was never considered by their care team in 39% of patients; in cases where transplant had been considered, 25% of patients who died without listing had refused LTx [6]. No studies to date have examined physicians’ knowledge, attitudes and behaviors surrounding referral of patients with CF for LTx evaluation. Thus, the objective of the current study was to elucidate potential barriers to referral of patients with CF for LTx evaluation through investigation of the perspectives of expert CF physicians in the US. We hypothesized that the study would reveal patterns of referral/non-referral and identify potential targets for intervention to improve access to LTx among patients with CF.

## Methods

The study protocol was reviewed by the Institutional Review Board at the University of Washington and was determined to afford Minimal Risk to subjects (physicians) (IRB #48957). A waiver of written informed consent was obtained; subjects were consented in the email introducing the survey and provided implicit consent when they followed the email link to the online survey.

### Data collection and study population

A 15-question investigator-designed online anonymous survey was created focused on referral of adults (≥18 years) with CF for LTx evaluation. The questions addressed CFF-care center characteristics, physician experience, indications/contraindications to referral for LTx evaluation, and opinions regarding the benefit of LTx in patients with CF (see Additional file 1 for survey items). The online survey was piloted with physicians who care for patients with CF at the University of Washington for feasibility. Pilot respondents found that the 15-question survey took fewer than 10 min to complete and was easily understood; pilot responses were not included in the final data analysis. The survey was formatted in SurveyMonkey® and subsequently distributed via email by the CFF to the center directors, program directors and affiliate directors of all CFF-accredited care centers in the US. Each CFF-care center received one survey for each of its program types (Adult, Affiliate, and Pediatric). As both Affiliate and Pediatric CF programs follow pediatric and adult patients albeit in varied proportions, they were included in the study to complete questions focused only on their adult patients. Surveys were completed between January 28, 2015 and February 20, 2015. Results were provided to the University of Washington investigators in an anonymous aggregated dataset, and were analyzed using STATA 13.1 (StataCorp LP, College Station, Texas).

### Statistical analyses

Descriptive statistics were used to describe the CFF-care center characteristics, physicians, and responses to lung transplant-related questions. The data was stratified by program type (Adult, Affiliate, Pediatric), CF physician experience (post-hoc dichotomization: <15 years versus ≥ 15 years), and distance from LTx center (direct affiliation [0 min distance] versus not directly affiliated [<30/30–75/75–150/>150 min driving distance]), and further analysis was performed on these subgroups.

The size of a CF center was estimated based on the number of adult CF patients cared for each year (entered as a “free text” number) and then categorized *a priori* into four groups (group1: <50 patients, group 2: 50–99 patients, group 3: 100–199 patients, group 4: ≥200 patients). The number of adult CF patients referred annually for lung transplant evaluation was also entered as a “free text” number; if a respondent provided a range (e.g. 0–5), then the mid-point of the range was used (rounded up to the nearest whole number) as the actual number referred per year. The percentage of adult CF patients referred annually was then calculated by dividing the number of patients referred by the absolute number of adult patients cared for each year. Respondents were asked to report the United Network for Organ Sharing (UNOS) region for their program and the proportion of respondents in each UNOS region was compared to the proportion of all CFF-accredited programs in each UNOS region in order to assess for a geographic response bias.

Chi-square analysis was used to evaluate for differences by program type (Adult, Affiliate, Pediatric) in distance from lung transplant center, years in independent CF practice, Lung Allocation Score (LAS) use, and patient-related reasons to defer referral for LTx evaluation. Multinomial (polytomous) regression was used to model differences in: model 1–program size and proportion of adult patients referred per year; model 2–indications that would trigger referral for LTx evaluation; model 3–colonization with specific organisms that would prevent referral for LTx evaluation; model 4–comorbidities that would prevent referral for LTx evaluation.

## Results

The survey was sent to 127 Pediatric Programs, 119 Adult Programs, and 86 CFF-Affiliated Programs. There were 114/332 (34%) total responses to the survey (Table 1). The response rates differed by Program type: 57/119 (48%) Adult Programs, 12/86 (14%) Affiliate Programs and 43/127 (34%) Pediatric Programs. Two respondents did not report their Program type. The number of years of independent practice of CF did not vary among Program types (*p* = 0.083); however, with dichotomization of CF physician experience (< or ≥15 years), Adult Programs have fewer respondents with ≥ 15 years of experience (*p* = 0.008). Respondents were evenly geographically distributed across the 11 UNOS transplant regions (Additional file 1: Figure S1). Respondents from Adult Programs were more likely to be directly affiliated with a lung transplant center than respondents from Affiliate or Pediatric Programs (*p* = 0.009) (Table 1); the distance from a lung transplant center was significantly different between Program types (*p* = 0.014). Adult Programs were larger than Affiliate and Pediatric Programs (*p* < 0.001), but there was no significant difference in the percentage of adult patients referred for LTx evaluation each year (multinomial regression data shown in Additional file 1).

**Table 1:** Demographics of respondents to surveys, grouped by Program type

	All Respondents^a^ *N* = 114	Adult Programs *N* = 57	Affiliate Programs *N* = 12	Pediatric Programs *N* = 43
Program size, *n* (%)^b^				
< 50 adult patients	38 (33%)	4 (7%)	11 (92%)	22 (51%)
50–99 adult patients	31 (27%)	18 (32%)	0	13 (30%)
100–199 adult patients	27 (24%)	21 (37%)	1 (8%)	5 (12%)
≥ 200 adult patients	17 (15%)	13 (23%)	0	3 (7%)
Percent referred/year^c^, Median (IQR)	3.5% (2.0%–6.4%)	3.2% (2.5%–5.8%)	5.0% (3.3%–7.5%)	3.3% (0–10.0%)
Distance to lung transplant center, *n* (%)				
Direct affiliation	39 (34%)	27 (47%)	2 (17%)	9 (21%)
< 30 min	16 (14%)	6 (11%)	1 (8%)	9 (21%)
30–75 min	18 (16%)	10 (18%)	0	8 (19%)
75–150 min	16 (14%)	7 (12%)	3 (25%)	5 (12%)
> 150 min	25 (22%)	7 (12%)	6 (50%)	12 (28%)
Years independently practicing CF medicine, *n* (%)				
< 5 years	13 (11%)	9 (16%)	0	3 (7%)
5 to < 10 years	19 (17%)	13 (23%)	2 (17%)	4 (9%)
10 to < 15 years	21 (18%)	12 (21%)	1 (8%)	7 (16%)
≥ 15 years	61 (54%)	23 (40%)	9 (75%)	29 (67%)

*IQR* interquartile range, *CF* cystic fibrosis

^a^Two respondents did not report Program type

^b^One adult Program did not report size of the Program

^c^Calculated by division of number of referrals by total number of adult patients followed by program(s)

Of the respondents, 107 (94%) stated that they would refer an adult patient for LTx evaluation if the patient’s lung function reached FEV_1_ < 30% predicted, 90 (79%) respondents would refer in the setting of a rapid decline in FEV_1_ and 110 (96%) respondents use at least one of those FEV_1_ criteria as a trigger for referral. Only approximately half (62/114, 54%) would refer a patient with a diagnosis of pulmonary hypertension (Table 2). Based on multinomial regression, respondents from a Pediatric Program were more likely than Adult Program respondents to choose a rapid decline in FEV_1_ as a trigger for LTx referral (*p* = 0.040). Other potential indications were not statistically different between Program types (Additional file 1: Table S1). Only 17/114 (15%) respondents reported considering the LAS prior to referral of patients, and rates of use were not significantly different between Program types (*p* = 0.125). In relation to patient outcomes, 100 (88%) and 103 (90%) respondents reported that LTx improves survival and quality of life, respectively, for patients with CF who undergo the procedure.

**Table 2:** Indications that would **trigger referral** for lung transplant evaluation, by Program type

	All Respondents^a^ *N* = 114	Adult Programs *N* = 57	Affiliate Programs *N* = 12	Pediatric Programs *N* = 43
	*n* (%)	*n* (%)	*n* (%)	*n* (%)
FEV_1_ < 30% predicted	107 (94%)	53 (93%)	12 (100%)	40 (93%)
NPPV for hypercapnia	96 (84%)	51 (90%)	11 (92%)	33 (77%)
Rapid decline in FEV_1_	90 (79%)	40 (70%)	10 (83%)	38 (88%)
Hemoptysis not controlled by embolization	75 (66%)	35 (61%)	9 (75%)	29 (67%)
Supplemental oxygen	64 (56%)	29 (51%)	9 (75%)	26 (61%)
Pulmonary hypertension	62 (54%)	34 (60%)	9 (75%)	19 (44%)
Increasing frequency pulmonary exacerbations	57 (50%)	25 (44%)	7 (58%)	24 (56%)
Refractory/recurrent pneumothorax	53 (47%)	26 (46%)	9 (75%)	18 (42%)
Pulmonary exacerbation with ICU admission	38 (33%)	16 (28%)	4 (33%)	18 (42%)
Skipped question^b^	2 (2%)	1 (2%)	0	1 (2%)

*FEV*_*1*_ Forced expiratory volume in 1 s, *NPPV* Noninvasive Positive Pressure Ventilation, *ICU* intensive care unit

^a^Two respondents did not report Program type

^b^Assumption: none of these would trigger referral for lung transplant evaluation

**Fig. 1:**
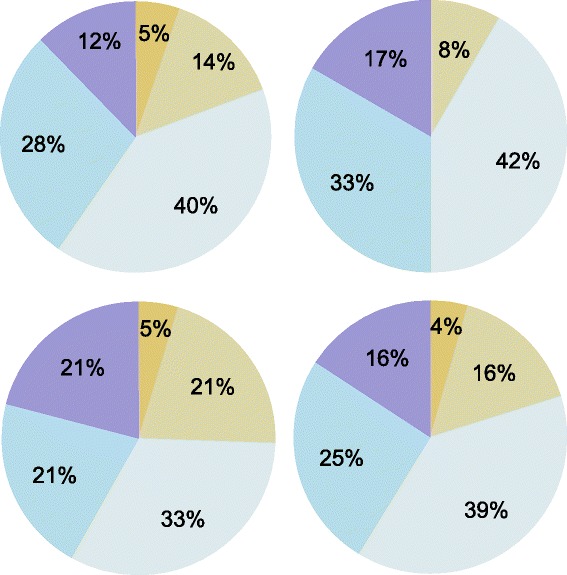
Proportion of physicians indicating primary patient-related reason to defer transplant referral is **patient preference**, by Program type. Panels show proportion of survey respondents who indicated primary patient-related reason to defer transplant referral is **patient preference**, by Program type: Adult Program (*top left*), Affiliate Program (*top right*), Pediatric Program (*bottom left*), All Respondents (*bottom right*)

**Fig. 2:**
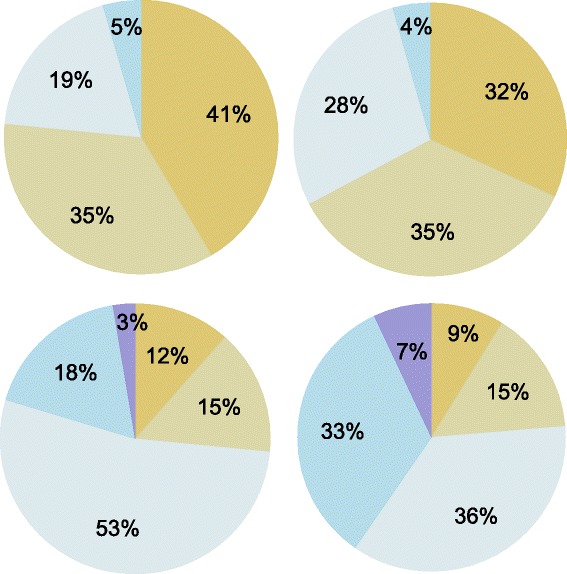
Proportion of physicians who indicated patient-related reasons to defer transplant referral. Panels show proportion of survey respondents who identified the following patient-related reasons to defer lung transplant referral: patient insurance (*top left*), patient financial issues (*top right*), patient social issues (*bottom left*), and patient adherence (*bottom right*)

Physicians were asked about potential contraindications that would prevent referral to a LTx center. The questions were grouped into three categories: colonization with specific organisms; co-morbidities; and patient-related factors. For these, 78 (68%) respondents indicated that colonization with a specific organism (*Burkholderia cenocepacia for a majority of respondents*) would prevent referral for LTx evaluation (Table 3). Based on multinomial regression, respondents from a Pediatric Program were less likely than Adult Program respondents to choose *B. cenocepacia* as a potential contraindication that would prevent LTx referral (*p* = 0.005) (Additional file 1: Table S2), which may be related to a decreased prevalence of *B. cenocepacia* in the Pediatric Programs [7]. With regards to co-morbidities preventing referral for LTx, 102 (89%) responded, and the most frequent ones were tissue diagnosis of cancer, end-stage renal disease (ESRD) requiring dialysis and inadequate nutritional status (e.g. body mass index, BMI <18) (Table 4). Based on multinomial regression, respondents from a Pediatric Program were less likely than Adult Program respondents to choose end-stage liver disease (ESLD) (*p* = 0.038), ESRD (*p* = 0.031), and depression (*p* = 0.032) as potential contraindications that would prevent LTx referral (Additional file 1: Table S3). Patient preference not to undergo LTx was “often” or “always” the primary patient-related reason to defer referral for LTx evaluation for 47/114 (41%) of respondents (Fig. 1) and was consistent across Program types (*p* = 0.837). Patient financial or insurance issues were “rarely” or “never” the primary patient-related reason to defer referral for LTx evaluation for a majority of respondents (Fig. 2). Chi-square analysis revealed that patient financial issues were more frequently the primary patient-related reason to defer referral for LTx evaluation among Adult Program respondents (*p* = 0.048). The free text “other” option was utilized by 15/114 (13%) of respondents to describe patient-related issues that would prevent referral. The “other” patient-related responses included: patient refusal/preference, non-adherence, tobacco/drug use, nutritional status, and financial issues.

**Table 3:** Colonization with specific organisms that would **prevent referral** for lung transplant evaluation, grouped by Program type

	All Respondents^a^ *N* = 114	Adult Programs *N* = 57	Affiliate Programs *N* = 12	Pediatric Programs *N* = 43
	*n* (%)	*n* (%)	*n* (%)	*n* (%)
*Burkholderia cenocepacia*	63 (55%)	37 (65%)	7 (58%)	17 (40%)
*Mycobacterium abscessus*	25 (22%)	10 (18%)	4 (33%)	11 (26%)
*Burkholderia cepacia* complex	24 (21%)	15 (26%)	2 (17%)	7 (16%)
*Mycobacterium avium* complex (MAC)	6 (5%)	3 (5%)	0	3 (7%)
Multidrug resistant bacteria^b^	3 (3%)	1 (2%)	0	2 (5%)
*Aspergillus fumigatus*	1 (1%)	1 (2%)	0	0
Skipped question^c^	36 (32%)	16 (28%)	3 (25%)	17 (40%)

^a^Two respondents did not report Program type

^b^Multidrug resistant bacteria, including: *Pseudomonas aeruginosa, Staphylococcus aureus, Stenotrophomonas maltophilia, Alcaligenes xylosoxidans*

^c^Assumption: none of these would prevent referral for lung transplant evaluation

**Table 4:** Comorbidities that would **prevent referral** for lung transplant evaluation, grouped by Program type

	All Respondents^a^ *N* = 114	Adult Programs *N* = 57	Affiliate Programs *N* = 12	Pediatric Programs *N* = 43
	*n* (%)	*n* (%)	*n* (%)	*n* (%)
Tissue diagnosis of cancer	74 (65%)	39 (68%)	9 (75%)	26 (61%)
CF-related ESRD requiring dialysis	50 (44%)	23 (40%)	6 (50%)	21 (49%)
Inadequate nutritional status (e.g. BMI <18)	33 (29%)	18 (32%)	4 (33%)	9 (21%)
CF-related liver cirrhosis	18 (16%)	13 (23%)	0	5 (12%)
CF-related diabetes, poorly controlled	18 (16%)	11 (19%)	2 (17%)	5 (12%)
Depression, anxiety	10 (9%)	3 (5%)	1 (8%)	6 (14%)
Pulmonary hypertension	1 (1%)	0	1 (8%)	0
Osteoporosis	1 (1%)	1 (2%)	0	0
CF-related sinus disease, extensive	0	0	0	0
Gastro-esophageal reflux disease	0	0	0	0
Other (free text response)	31 (27%)	19 (33%)	3 (25%)	9 (21%)
Skipped question^b^	12 (11%)	5 (9%)	0	7 (16%)

*CF* cystic fibrosis, *ESRD* end-stage renal disease, *BMI* body mass index

^a^Two respondents did not report Program type

^b^Assumption: none of these would prevent referral for lung transplant evaluation

Analyses were also performed on the subgroups defined by distance from LTx center and CF physician experience to identify associations with Program types, and causes of deferred LTx evaluation and detailed results can be found in the supplemental materials (Additional file 1: Tables S4–S9).

## Discussion

Our national survey of CF physicians in the US herein identified a number of associations and explanations for deferred referral of patients with CF for LTx evaluation that merit further study. Nearly all physicians who responded to our survey reported that they use the FEV_1_ (either <30% or a rapid decline) as a trigger for referral for LTx evaluation. It is clear that these expert CF physicians recognize the lung function criteria for LTx referral. Observational data suggests that survival among patients with CF and FEV_1_ < 30% is improving over time [8–10], and a national cohort study of patients in the US CFF Patient Registry, 2003–2013, demonstrated a median transplant-free survival of 6.6 years after FEV_1_ < 30%. With this observation of increasing survival in patients with CF and severe lung disease (FEV_1_ < 30%), we believe that this may explain recent findings of low referral rates among patients with CF and FEV_1_ < 30% in the CFF Patient Registry [4, 5] regardless of current International Society for Heart and Lung Transplantation (ISHLT) guidelines [11].

The timing of referral for LTx evaluation is an aspect of the “art” of caring for patients with CF, as individualized survival estimates are challenging. Our study thus highlights important practices among expert CF physicians. The annual median percentage of adult patients at each center referred for LTx evaluation was 3.5% and this varied little in subgroup analyses of Program type, distance from LTx center and CF physician experience. Nearly all respondents report that FEV_1_ < 30% predicted would trigger a referral for LTx evaluation, but the proportion of CF physicians that consider supplemental oxygen use, pulmonary hypertension, or the frequency/severity of pulmonary exacerbations is much lower. All of the indications listed in Table 2 are within the ISHLT guidelines for referral for LTx evaluation for patients with CF, and certain ones are indications to list for LTx (not just indications for referral) [11]. Additionally, very few respondents report considering the LAS prior to referral for LTx evaluation; the LAS was adopted in May 2005 in the US in an attempt to maximize net benefit of transplant by integrating a patient’s waitlist urgency and 1-year post-transplant survival [12]. The LAS is also utilized for LTx allocation in Germany as of December 2011 [13]. Knowing the components of the LAS that lead to a higher score (e.g. supplemental oxygen requirement, decreased 6-min walk distance, elevated pulmonary artery pressure) could inform a referring physician’s decision about the timing of referral in the US. Higher LAS scores are associated with worsened survival among patients with CF [14, 15] and the LAS incorporates some of the criteria in the ISHLT guidelines for listing for lung transplantation–elements that are relevant even in countries that do not utilize the LAS. It is possible that CF physicians are focused on lung function parameters in the guidelines (e.g. FEV_1_ < 30% predicted) instead of other important factors considered in the LTx listing process (e.g. components of the LAS, or frequency/severity of pulmonary exacerbations). This focus on lung function may be related to a lack of data about other parameters (screening for pulmonary hypertension is not within the CFF guidelines; 6-min walk tests are not routinely recommended), but focusing only on lung function could lead to missed opportunities for referral and provides an opportunity for education about current LTx practices. Alternatively, CF physicians may have adjusted their referral practices (at least with respect to lung function parameters) as their clinical experience has revealed patients do not seem ready for a LTx evaluation despite meeting current lung function guidelines for referral. Unfortunately, many patients with FEV_1_ < 30% die without LTx, and increasing referral within this population is critical [5, 6].

Additionally, we observed that a majority of respondents identified *Burkholderia cenocepacia* as a contraindication to LTx referral. Multiple studies, including a recent study of Canadian patients with CF, have demonstrated that survival is significantly shortened in patients infected with *B. cenocepacia* who undergo LTx [16–19]. Most LTx centers in the US consider *B. cenocepacia* to be an absolute contraindication to LTx. Furthermore, in regard to comorbidities, respondents who were directly affiliated with a LTx center were more likely to identify ESLD as a reason to defer referral, which may be based on the experience that liver-lung transplantation is exceedingly rare because of the complexity of the combined surgeries. Respondents directly affiliated with a LTx center were also more likely to identify inadequate nutritional status as a reason to defer referral, which may be based on the experience that LTx programs often ask the primary CF center to optimize nutritional status prior to referral. However, it is important to note that many comorbidities are relative contraindications and early referral may allow specialists at LTx centers to intervene on modifiable comorbidities. Finally, CF physicians’ perceptions of the most frequent patient-related reasons to defer referral included “patient preference not to undergo lung transplantation” and patient non-adherence. This raises the question of whether patients understand the risks and benefits of LTx prior to referral and the consequences of their decision to defer referral.

Our study has important limitations to consider. As our overall response rate was low, and at differential rates between programs, selection bias may have occurred. However, we had a nearly 50% response rate amongst CF physicians from Adult Programs who care for the majority of our study population of interest. Most recipients of LTx in CF are over 30 years old [20]. Respondents from the Affiliate and Pediatric programs care for fewer adult patients with CF, and this may have impacted their survey responses. The response bias may have been directed towards those with greater experience and knowledge in CF and transplant care and thus variability of responses observed in the study may have been attenuated relative to the CF care community at large. A number of statistical analyses were conducted of the survey data leading to a potential concern about multiple testing. We used multinomial regression to reduce this concern, but false positive statistical associations are possible. Although our study was conducted in the US, CF care in the US aims to adhere to evidence-based standards and the LTx referral guidelines are international recommendations, and thus our survey may have generalizability to the CF global community. Additionally, though a responder bias cannot be excluded, within the US, we did not identify any significant regional differences in response rates. Despite the limitations, this was the first large multicenter survey study to examine the attitudes, behaviors, and LTx referral patterns of CF physicians to inform future studies in this important area of care.

## Conclusions

The decision to pursue lung transplantation is a complex balance of patient preference and physician judgment of medical indication and patient candidacy. Our study indicates that barriers to referral may be related to patient preferences, clinical characteristics, or physician knowledge regarding non-lung function-based recommendations related to the timing of referral and listing for LTx. Interventions to educate CF physicians (both adult and pediatric patient providers) about the non-lung function-based factors that impact listing for LTx should be studied and implemented. Further exploration of physician-level and CF patient-level barriers to timely LTx referral is warranted.

## Additional file

Additional file 1:
**Methods**. Questionnaire for CFF-Accredited Program Directors. **Results**. **Figure S1**. Regional Distribution of Programs. **Table S1**. Multinomial regression of indications that would **trigger referral** for lung transplant evaluation, by Program type. **Table S2**. Multinomial regression of responses for colonization with specific organisms that would **prevent referral** for lung transplant evaluation, grouped by Program type. **Table S3**. Multinomial regression of responses for comorbidities that would **prevent referral** for lung transplant evaluation, grouped by Program type**. Table S4**. Demographics of respondents to surveys, grouped by distance from lung transplant (LTx) center. **Table S5**. Indications that would **trigger referral** for lung transplant evaluation, by distance from lung transplant (LTx) center. **Table S6**. Colonization with specific organisms that would **prevent referral** for lung transplant evaluation, grouped by distance from lung transplant (LTx) center. **Table S7**. Comorbidities that would **prevent referral** for lung transplant evaluation, grouped by distance from lung transplant (LTx) center. **Table S8**. Demographics of respondents to surveys, grouped by cystic fibrosis (CF) physician experience. **Table S9**: Indications that would **trigger referral** for lung transplant evaluation, by cystic fibrosis (CF) physician experience. **Table S10**. Colonization with specific organisms that would **prevent referral** for lung transplant evaluation, grouped by cystic fibrosis (CF) physician experience. **Table S11**. Comorbidities that would **prevent referral** for lung transplant evaluation, grouped by cystic fibrosis (CF) physician experience. Chi square analysis and multinomial regression results. (DOCX 50 kb)

## Abbreviations

BMI: Body mass index
CF: Cystic fibrosis
CFF: Cystic Fibrosis Foundation
CFRD: CF-related diabetes
ESLD: End-stage liver disease
ESRD: End-stage renal disease
ISHLT: International Society for Heart and Lung Transplantation
LAS: Lung Allocation Score
LTx: Lung transplantation
UNOS: United Network for Organ Sharing
