# Supplementary material for: Cystic fibrosis physicians’ perspectives on the timing of referral for lung transplant evaluation: a survey of physicians in the United States
Source: BMC Pulm Med. 2017 Jan 19;17:21. doi: 10.1186/s12890-017-0367-9 (PMC5248524; doi:10.1186/s12890-017-0367-9)
Supplement: Additional file 1: — Methods. Questionnaire for CFF-Accredited Program Directors. Results. Figure S1. Regional Distribution of Programs. Table S1. Multinomial regression of indications that would trigger referral for lung transplant evaluation, by Program type. Table S2. Multinomial regression of responses for colonization with specific organisms that would prevent referral for lung transplant evaluation, grouped by Program type. Table S3. Multinomial regression of responses for comorbidities that would prevent referral for lung transplant evaluation, grouped by Program type. Table S4. Demographics of respondents to surveys, grouped by distance from lung transplant (LTx) center. Table S5. Indications that would trigger referral for lung transplant evaluation, by distance from lung transplant (LTx) center. Table S6. Colonization with specific organisms that would prevent referral for lung transplant evaluation, grouped by distance from lung transplant (LTx) center. Table S7. Comorbidities that would prevent referral for lung transplant evaluation, grouped by distance from lung transplant (LTx) center. Table S8. Demographics of respondents to surveys, grouped by cystic fibrosis (CF) physician experience. Table S9: Indications that would trigger referral for lung transplant evaluation, by cystic fibrosis (CF) physician experience. Table S10. Colonization with specific organisms that would prevent referral for lung transplant evaluation, grouped by cystic fibrosis (CF) physician experience. Table S11. Comorbidities that would prevent referral for lung transplant evaluation, grouped by cystic fibrosis (CF) physician experience. Chi square analysis and multinomial regression results. (DOCX 50 kb) [file 12890_2017_367_MOESM1_ESM.docx]

**Additional file 1**

Cystic fibrosis physicians’ perspectives on the timing of referral for lung transplant evaluation:

A survey of physicians in the United States

Kathleen J. Ramos, MD

Ranjani Somayaji, MD

Erika D. Lease, MD

Christopher H. Goss, MD, MSc, FCCP

Moira L. Aitken, MD

**Methods**

**Questionnaire for CFF-Accredited Program Directors**

1. Choose your CF program type: (drop down menu: adult/pediatric/affiliate)
2. Approximately how many patients (age >18 years) does your CF center care for annually? (fill in)
3. Approximately how many patients (age >18 years) are referred for lung transplant evaluation annually? (fill in)
4. What is your estimate of the distance to your nearest lung transplant center? (drop down menu with a range in driving time: 0, <30 minutes, 30-75 minutes, 75-150 minutes, >150 minutes) Note: please enter “0” if directly affiliated with a lung transplant center
5. Which UNOS transplant region do you belong to? (drop down menu listing region: states)

Region 1: Connecticut, Maine, Massachusetts, New Hampshire, Rhode Island, Eastern Vermont

Region 2: Delaware, District of Columbia, Maryland, New Jersey, Pennsylvania, West Virginia, Northern Virginia

Region 3: Alabama, Arkansas, Florida, Georgia, Louisiana, Mississippi, Puerto Rico

Region 4: Oklahoma, Texas

Region 5: Arizona, California, Nevada, New Mexico, Utah

Region 6: Alaska, Hawaii, Idaho, Montana, Oregon, Washington

Region 7: Illinois, Minnesota, North Dakota, South Dakota, Wisconsin

Region 8: Colorado, Iowa, Kansas, Missouri, Nebraska, Wyoming

Region 9: New York, Western Vermont

Region 10: Indiana, Michigan, Ohio

Region 11: Kentucky, North Carolina, South Carolina, Tennessee, Virginia

1. How many years have you been independently practicing in CF care? (drop down menu: <5 years, 5 to <10 years, 10 to <15 years, >15 years)
2. Which of the following indications would trigger referral for lung transplant evaluation at your CF center (check all that apply):
   - FEV1 <30% predicted
   - Rapid decline in FEV1
   - Pulmonary disease exacerbation requiring ICU admission
   - Increasing frequency of pulmonary exacerbations requiring antibiotics
   - Refractory or recurrent pneumothorax
   - Recurrent hemoptysis not controlled by embolization
   - Pulmonary hypertension
   - Supplemental oxygen requirement
   - Non-invasive mechanical ventilation for hypercapnia
   - Other:______________________________________________
3. When considering referral for transplant evaluation, do you consider the patient’s lung allocation score (LAS) prior to initiating a referral? (yes/no/I don’t know)
4. Colonization with which of the following potential organisms would prevent referral for lung transplant evaluation at your CF center (check all that apply):
   - Multidrug resistant organisms (*Pseudomonas aeruginosa, Staphylococcus aureus, Stenotrophomonas maltophilia, Alcaligenes xylosoxidans*)
   - *Aspergillus fumigatus*
   - *Burkholderia cenocepacia*
   - *Burkholderia cepacia* complex
   - *Mycobacterium abscessus*
   - *Mycobacterium avium* complex (MAC)
5. Which of the following potential comorbidity-related contraindications would prevent referral for lung transplant evaluation at your CF center (check all that apply):
   - CF-related liver cirrhosis
   - CF-related end-stage kidney disease requiring dialysis
   - CF-related diabetes, poorly controlled
   - CF-related sinus disease, extensive
   - Inadequate nutritional status (i.e. BMI <18)
   - Osteoporosis
   - GERD
   - Pulmonary hypertension
   - Tissue diagnosis of cancer
   - Depression/Anxiety
   - Other:__________________________________________
6. How often are the following patient-related contraindications the primary reason to defer referral in your patient population (button matrix: 1 = never/2 = rarely/3 = sometimes/4 = often/ 5 = always):
   - Patient preference not to undergo lung transplantation:
   - Poor adherence:
   - Patient insurance issues:
   - Patient financial issues:
   - Lack of social support:
   - Other:_________________________________________
7. What percentage of post-transplant CF patients return to your core CF clinic for routine care? (drop down menu in 10% increments)
8. What percentage of post-transplant CF patients are maintained in the CF Foundation patient registry? (drop down menu in 10% increments)
9. In your opinion, does lung transplantation improve survival for the majority of CF patients who undergo transplant? (yes/no/I don’t know)
10. In your opinion, does lung transplantation improve quality of life for the majority of CF patients who undergo transplant? (yes/no/I don’t know)

**Results**

Figure S1. In order to assess the potential for a regional bias of response to the survey we are displaying the proportion (percentage) of CFF-accredited Programs in each of the 11 UNOS regions (red bars) and the percentage of respondents’ Programs in each of the 11 UNOS regions (blue bars). The geographic distribution of respondents’ Programs appears to mirror the geographic distribution of all CFF-accredited Programs.

Multinomial regression (model 1) reveals a statistically significant difference in the group size of the different Program types; RR of group size was 0.07 for Affiliate Programs (p <0.001) and 0.33 for Pediatric Programs (p <0.001), with Adult Programs as the reference. There was not, however, a significant difference in the median percent referred per year by Program type, adjusted for group size (p>0.05 for both Affiliate and Pediatric Programs).

| Table S1. Multinomial regression of indications that would **trigger referral** for lung transplant evaluation, by Program type | | |
| --- | --- | --- |
|  | **Affiliate Programs***  N = 12 | **Pediatric Programs***  N = 43 |
|  | RR (95% CI) | RR (95% CI) |
| **FEV_1_<30% predicted** | 9.51 x10^5^ (0, undefined) | 0.62 (0.10, 3.73) |
| **NPPV for hypercapnia** | 0.22 (0.01, 4.24) | 0.34 (0.7, 1.55) |
| **Rapid decline in FEV_1_** | 1.73 (0.28, 10.59) | 3.87 (1.07, 14.04) ^†^ |
| **Hemoptysis not controlled by embolization** | 0.91 (0.14, 5.72) | 1.64 (0.57, 4.73) |
| **Supplemental oxygen** | 2.42 (0.48, 12.13) | 1.91 (0.71, 5.13) |
| **Pulmonary hypertension** | 2.12 (0.36, 12.47) | 0.46 (0.16,1.30) |
| **Increasing frequency pulmonary exacerbations** | 0.89 (0.20, 3.97) | 0.99 (0.36, 2.69) |
| **Refractory/recurrent pneumothorax** | 3.84 (0.63, 23.28) | 0.62 (0.22, 1.72) |
| **Pulmonary exacerbation with ICU admission** | 0.62 (0.13, 2.92) | 1.70 (0.60, 4.83) |
| p-values >0.05 (non-significant) unless otherwise specified. STATA determines that a confidence interval is “undefined” when standard errors are questionable.  * Reference group is Adult Programs  † p-value 0.040  FEV_1_ = Forced expiratory volume in 1 second; NPPV = Noninvasive Positive Pressure Ventilation; ICU = intensive care unit | | |

| Table S2. Multinomial regression of responses for colonization with specific organisms that would **prevent referral** for lung transplant evaluation, grouped by Program type | | |
| --- | --- | --- |
|  | **Affiliate Programs***  N = 12 | **Pediatric Programs***  N = 43 |
|  | RR (95% CI) | RR (95% CI) |
| ***Burkholderia cenocepacia*** | 0.64 (0.17, 2.43) | 0.28 (0.12, 0.68) † |
| ***Mycobacterium abscessus*** | 3.27 (0.77, 13.97) | 1.87 (0.60, 5.85) |
| ***Burkholderia cepacia* complex** | 0.73 (0.14, 3.94) | 0.70 (0.23, 2.12) |
| ***Mycobacterium avium* complex (MAC)** | 2.44x10^-7^ (0, undefined) | 2.01 (0.23, 17.42) |
| **Multidrug resistant bacteria ^ⱡ^** | 0.84 (0, undefined) | 2.79x10^10^ (0, undefined) |
| ***Aspergillus fumigatus*** | 0.20 (0, undefined) | 8.82x10^-16^ (0, undefined) |
| p-values >0.05 (non-significant) unless otherwise specified. STATA determines that a confidence interval is “undefined” when standard errors are questionable.  * Reference group is Adult Programs  † p-value 0.005  **^ⱡ^** Multidrug resistant bacteria, including: *Pseudomonas aeruginosa, Staphylococcus aureus, Stenotrophomonas maltophilia, Alcaligenes xylosoxidans* | | |

| Table S3. Multinomial regression of responses for comorbidities that would **prevent referral** for lung transplant evaluation, grouped by Program type | | |
| --- | --- | --- |
|  | **Affiliate Programs***  N = 12 | **Pediatric Programs***  N = 43 |
|  | RR (95% CI) | RR (95% CI) |
| **Tissue diagnosis of cancer** | 1.21 (0.26, 5.62) | 0.58 (0.23, 1.49) |
| **CF-related ESRD requiring dialysis** | 2.39 (0.57, 10.13) | 2.99 (1.11, 8.11) † |
| **Inadequate nutritional status (i.e. BMI <18)** | 2.23 (0.50, 9.94) | 0.67 (0.22, 2.01) |
| **CF-related liver cirrhosis** | 1.63x10^-7^ (0, undefined) | 0.16 (0.06, 0.92) † |
| **CF-related diabetes, poorly controlled** | 0.38 (0.04, 3.92) | 0.54 (0.14, 2.02) |
| **Depression, anxiety** | 1.62x10^-6^ (0, undefined) | 6.69 (1.18, 37.87) † |
| **Pulmonary hypertension** | 2.07x10^16^ (0, undefined) | 0.14 (0, undefined) |
| **Osteoporosis** | 2.58x10^-8^ (0, undefined) | 8.76x10-8 (0, undefined) |
| **CF-related sinus disease, extensive ^ⱡ^** | 1 (omitted) | 1 (omitted) |
| **Gastro-esophageal reflux disease ^ⱡ^** | 1 (omitted) | 1 (omitted) |
| p-values >0.05 (non-significant) unless otherwise specified. STATA determines confidence interval is “undefined” when standard errors are questionable.  * Reference group is Adult Programs  † CF-related liver cirrhosis (p-value 0.038); CF-related ESRD (p-value 0.031); Depression, anxiety (p-value 0.032)  ^ⱡ^ RR and 95% CI omitted due to colinearity  CF = cystic fibrosis, ESRD = end-stage renal disease, BMI = body mass index | | |

| Table S4. Demographics of respondents to surveys, grouped by distance from lung transplant (LTx) center | | | |
| --- | --- | --- | --- |
|  | **All Respondents**  N = 114 | **Directly Affiliated with LTx Center**  N = 39 | **Not Directly Affiliated with LTx Center**  N = 75 |
| **Program type**, n (%) |  |  |  |
| Adult Program | 57 (50%) | 27 (69%) | 30 (40%) |
| Affiliate Program | 12 (11%) | 2 (5%) | 10 (13%) |
| Pediatric Program | 43 (38%) | 9 (23%) | 34 (45%) |
| No Program Type* | 2 (2%) | 1 (3%) | 1 (1%) |
| **Program size**, n (%) |  |  |  |
| < 50 adult patients | 38 (33%) | 5 (13%) | 33 (44%) |
| 50-99 adult patients | 31 (27%) | 7 (18%) | 24 (32%) |
| 100-199 adult patients | 27 (24%) | 13 (33%) | 14 (19%) |
| ≥ 200 adult patients | 17 (15%) | 14 (36%) | 3 (4%) |
| No Program Size **^†^** | 1 (1%) | 0 | 1 (1%) |
| **Distance to lung transplant center**, n (%) |  |  |  |
| Direct affiliation | 39 (34%) | 39 (100%) | 0 |
| < 30 minutes | 16 (14%) |  | 16 (21%) |
| 30-75 minutes | 18 (16%) |  | 18 (24%) |
| 75-150 minutes | 16 (14%) |  | 16 (21%) |
| > 150 minutes | 25 (22%) |  | 25 (33%) |
| **Percent referred/year**, Median (IQR) | 3.5%  (2.0% – 6.4%) | 3.1%  (2.0% - 5.5%) | 4.0%  (2.0% - 6.7%) |
| **Years independently practicing CF medicine**, n (%) |  |  |  |
| < 5 years | 13 (11%) | 6 (15%) | 7 (9%) |
| 5 to < 10 years | 19 (17%) | 3 (8%) | 16 (21%) |
| 10 to < 15 years | 21 (18%) | 11 (28%) | 10 (13%) |
| ≥ 15 years | 61 (54%) | 19 (49%) | 42 (56%) |
| * Two respondents did not report program type  ^†^ One adult program did not report size of the program  LTx = lung transplant, IQR = interquartile range, CF = cystic fibrosis | | | |

Based on Chi square analysis direct affiliation with a LTx center was not associated with: LAS use (p=0.057); years of CF experience (p=0.071); or a difference in any of the patient-related reasons [patient preference not to undergo LTx (p=0.541); adherence issues (p=0.355); insurance issues (p=0.303); financial issues (p=0.408); and social issues (p=0.404)] to defer referral for LTx evaluation. Based on multinomial regression, direct affiliation with a LTx center was not associated with indications that would trigger referral for LTx evaluation (p >0.05). Direct affiliation was associated with: Program type (Chi-square, p=0.009); increased likelihood that *B. cenocepacia* [RR 3.75 (95%CI 1.52, 9.27); p=0.004], ESLD [RR 6.12 (95% CI 1.53, 24.52; p=0.010], and inadequate nutritional status [RR 2.80 (95% CI 1.04, 7.55); p=0.042] would prevent referral; decreased likelihood that CFRD [RR 0.23 (95%CI 0.05, 0.97); p=0.046] would prevent referral.

| Table S5. Indications that would **trigger referral** for lung transplant evaluation, by distance from lung transplant (LTx) center | | | |
| --- | --- | --- | --- |
|  | **All Respondents**  N = 114 | **Directly Affiliated with LTx Center**  N = 39 | **Not Directly Affiliated with LTx Center**  N = 75 |
|  | n (%) | n (%) | n (%) |
| **FEV_1_ <30% predicted** | 107 (94%) | 36 (92%) | 71 (95%) |
| **NPPV for hypercapnia** | 96 (84%) | 33 (85%) | 63 (84%) |
| **Rapid decline in FEV_1_** | 90 (79%) | 32 (82%) | 58 (77%) |
| **Hemoptysis not controlled by embolization** | 75 (66%) | 26 (67%) | 49 (65%) |
| **Supplemental oxygen** | 64 (56%) | 22 (56%) | 42 (56%) |
| **Pulmonary hypertension** | 62 (54%) | 25 (64%) | 37 (49%) |
| **Increasing frequency pulmonary exacerbations** | 57 (50%) | 19 (49%) | 38 (51%) |
| **Refractory/recurrent pneumothorax** | 53 (47%) | 20 (51%) | 33 (44%) |
| **Pulmonary exacerbation with ICU admission** | 38 (33%) | 14 (36%) | 24 (32%) |
| **Skipped question^ⱡ^** | 2 (2%) | 1 (3%) | 1 (1%) |
| **^ⱡ^** Assumption: none of these would trigger referral for lung transplant evaluation  LTx = lung transplant; FEV_1_ = Forced expiratory volume in 1 second; NPPV = Noninvasive Positive Pressure Ventilation; ICU = intensive care unit | | | |

| Table S6. Colonization with specific organisms that would **prevent referral** for lung transplant evaluation, grouped by distance from lung transplant (LTx) center | | | |
| --- | --- | --- | --- |
|  | **All Respondents**  N = 114 | **Directly Affiliated with LTx Center**  N = 39 | **Not Directly Affiliated with LTx Center**  N = 75 |
|  | n (%) | n (%) | n (%) |
| ***Burkholderia cenocepacia*** | 63 (55%) | 29 (74%) | 34 (45%) |
| ***Mycobacterium abscessus*** | 25 (22%) | 9 (23%) | 16 (21%) |
| ***Burkholderia cepacia* complex** | 24 (21%) | 9 (23%) | 15 (20%) |
| ***Mycobacterium avium* complex (MAC)** | 6 (5%) | 1 (3%) | 5 (7%) |
| **Multidrug resistant bacteria^†^** | 3 (3%) | 3 (8%) | 0 |
| ***Aspergillus fumigatus*** | 1 (1%) | 1 (3%) | 0 |
| **Skipped question^ⱡ^** | 36 (32%) | 7 (18%) | 29 (39%) |
| ^†^ Multidrug resistant bacteria, including: *Pseudomonas aeruginosa, Staphylococcus aureus, Stenotrophomonas maltophilia, Alcaligenes xylosoxidans*  **^ⱡ^** Assumption: none of these would prevent referral for lung transplant evaluation  LTx = lung transplant | | | |

| Table S7. Comorbidities that would **prevent referral** for lung transplant evaluation, grouped by distance from lung transplant (LTx) center | | | |
| --- | --- | --- | --- |
|  | **All Respondents**  N = 114 | **Directly Affiliated with LTx Center**  N = 39 | **Not Directly Affiliated with LTx Center**  N = 75 |
|  | n (%) | n (%) | n (%) |
| **Tissue diagnosis of cancer** | 74 (65%) | 30 (77%) | 44 (59%) |
| **CF-related ESRD requiring dialysis** | 50 (44%) | 18 (46%) | 32 (43%) |
| **Inadequate nutritional status (i.e. BMI <18)** | 33 (29%) | 17 (44%) | 16 (21%) |
| **Other (free text response)** | 31 (27%) | 12 (31%) | 19 (25%) |
| **CF-related liver cirrhosis** | 18 (16%) | 11 (28%) | 7 (9%) |
| **CF-related diabetes, poorly controlled** | 18 (16%) | 4 (10%) | 14 (19%) |
| **Depression, anxiety** | 10 (9%) | 3 (8%) | 7 (9%) |
| **Pulmonary hypertension** | 1 (1%) | 0 | 1 (1%) |
| **Osteoporosis** | 1 (1%) | 1 (3%) | 0 |
| **CF-related sinus disease, extensive** | 0 | 0 | 0 |
| **Gastro-esophageal reflux disease** | 0 | 0 | 0 |
| **Skipped question^ⱡ^** | 12 (11%) | 3 (8%) | 9 (12%) |
| **^ⱡ^** Assumption: none of these would prevent referral for lung transplant evaluation  LTx = lung transplant; CF = cystic fibrosis, ESRD = end-stage renal disease, BMI = body mass index | | | |

| Table S8. Demographics of respondents to surveys, grouped by cystic fibrosis (CF) physician experience | | | |
| --- | --- | --- | --- |
|  | **All Respondents**  N = 114 | **CF Physician Experience <15 years**  N = 53 | **CF Physician Experience ≥15 years**  N = 61 |
| **Program type**, n (%) |  |  |  |
| Adult Program | 57 (50%) | 34 (64%) | 23 (38%) |
| Affiliate Program | 12 (11%) | 3 (6%) | 9 (15%) |
| Pediatric Program | 43 (38%) | 14 (16%) | 29 (48%) |
| No Program Type* | 2 (2%) | 2 (4%) | 0 |
| **Program size**, n (%) |  |  |  |
| < 50 adult patients | 38 (33%) | 11 (21%) | 27 (44%) |
| 50-99 adult patients | 31 (27%) | 19 (36%) | 12 (20%) |
| 100-199 adult patients | 27 (24%) | 11 (21%) | 16 (26%) |
| ≥ 200 adult patients | 17 (15%) | 12 (23%) | 5 (8%) |
| No Program Size **^†^** | 1 (1%) | 0 | 1 (2%) |
| **Distance to lung transplant center**, n (%) |  |  |  |
| Direct affiliation | 39 (34%) | 20 (38%) | 19 (31%) |
| < 30 minutes | 16 (14%) | 6 (11%) | 10 (16%) |
| 30-75 minutes | 18 (16%) | 6 (11%) | 12 (20%) |
| 75-150 minutes | 16 (14%) | 8 (15%) | 8 (13%) |
| > 150 minutes | 25 (22%) | 13 (25%) | 12 (20%) |
| **Percent referred/year**, Median (IQR) | 3.5%  (2.0% – 6.4%) | 3.5%  (2.0% - 6.25%) | 3.5%  (2.0% - 7.1%) |
| **Years independently practicing CF medicine**, n (%) |  |  |  |
| < 5 years | 13 (11%) | 13 (25%) | 0 |
| 5 to < 10 years | 19 (17%) | 19 (36%) | 0 |
| 10 to < 15 years | 21 (18%) | 21 (40%) | 0 |
| ≥ 15 years | 61 (54%) | 0 | 61 (100%) |
| * Two respondents did not report program type  ^†^ One adult program did not report size of the program  CF = cystic fibrosis; IQR = interquartile range | | | |

Increased CF physician experience (≥ 15 years) was not associated with: distance from a LTx center (Chi-square, p=0.642); direct affiliation with a LTx center (Chi-square, p=0.460); a difference in patient-related reasons [patient preference not to undergo LTx (Chi-square, p=0.221); adherence issues (Chi-square, p=0.179); insurance issues (Chi-square, p=0.308); financial issues (Chi-square, p=0.212); and social issues (Chi-square, p=0.906)] to defer referral for LTx evaluation; LAS use (Chi-square, p=0.718); comorbidities that would prevent referral (multinomial regression, p>0.05 for all covariates); median percentage of adult patients referred per year (multinomial regression, p=0.230). Increased CF physician experience (≥ 15 years) was associated with: Program type (Chi-square, p=0.008); Program size [RR 0.63 (95%CI 0.43, 0.91); p=0.013); increased likelihood that M. abscessuss colonization would prevent referral [RR 4.95 (95%CI 1.51, 16.28); p=0.008]; increased likelihood that a pulmonary exacerbation requiring ICU admission [RR 2.96 (95%CI 1.10, 7.95); p=0.031] or recurrent/refractory pneumothorax [RR 4.14 (95%CI 1.52, 11.28); p=0.005] would trigger referral; decreased likelihood that hemoptysis not controlled with embolization [RR 0.32 (95%CI 0.11, 0.91);p=0.032] would trigger referral.

| Table S9. Indications that would **trigger referral** for lung transplant evaluation, by cystic fibrosis (CF) physician experience | | | |
| --- | --- | --- | --- |
|  | **All Respondents**  N = 114 | **CF Physician Experience <15 years**  N = 53 | **CF Physician Experience ≥15 years**  N = 61 |
|  | n (%) | n (%) | n (%) |
| **FEV_1_ <30% predicted** | 107 (94%) | 52 (98%) | 55 (90%) |
| **NPPV for hypercapnia** | 96 (84%) | 43 (81%) | 53 (87%) |
| **Rapid decline in FEV_1_** | 90 (79%) | 42 (79%) | 48 (79%) |
| **Hemoptysis not controlled by embolization** | 75 (66%) | 36 (68%) | 39 (64%) |
| **Supplemental oxygen** | 64 (56%) | 23 (43%) | 41 (67%) |
| **Pulmonary hypertension** | 62 (54%) | 27 (51%) | 35 (57%) |
| **Increasing frequency pulmonary exacerbations** | 57 (50%) | 26 (49%) | 31 (51%) |
| **Refractory/recurrent pneumothorax** | 53 (47%) | 17 (32%) | 36 (59%) |
| **Pulmonary exacerbation with ICU admission** | 38 (33%) | 12 (23%) | 26 (43%) |
| **Skipped question^ⱡ^** | 2 (2%) | 0 | 2 (3%) |
| **^ⱡ^** Assumption: none of these would trigger referral for lung transplant evaluation  CF = cystic fibrosis; FEV_1_ = Forced expiratory volume in 1 second; NPPV = Noninvasive Positive Pressure Ventilation; ICU = intensive care unit | | | |

| Table S10. Colonization with specific organisms that would **prevent referral** for lung transplant evaluation, grouped by cystic fibrosis (CF) physician experience | | | |
| --- | --- | --- | --- |
|  | **All Respondents**  N = 114 | **CF Physician Experience <15 years**  N = 53 | **CF Physician Experience ≥15 years**  N = 61 |
|  | n (%) | n (%) | n (%) |
| ***Burkholderia cenocepacia*** | 63 (55%) | 30 (57%) | 33 (54%) |
| ***Mycobacterium abscessus*** | 25 (22%) | 6 (11%) | 19 (31%) |
| ***Burkholderia cepacia* complex** | 24 (21%) | 9 (17%) | 15 (25%) |
| ***Mycobacterium avium* complex (MAC)** | 6 (5%) | 3 (6%) | 3 (5%) |
| **Multidrug resistant bacteria^†^** | 3 (3%) | 0 | 3 (5%) |
| ***Aspergillus fumigatus*** | 1 (1%) | 0 | 1 (2%) |
| **Skipped question^ⱡ^** | 36 (32%) | 18 (34%) | 18 (30%) |
| ^†^ Multidrug resistant bacteria, including: *Pseudomonas aeruginosa, Staphylococcus aureus, Stenotrophomonas maltophilia, Alcaligenes xylosoxidans*  **^ⱡ^** Assumption: none of these would prevent referral for lung transplant evaluation  CF = cystic fibrosis | | | |

| Table S11. Comorbidities that would **prevent referral** for lung transplant evaluation, grouped by cystic fibrosis (CF) physician experience | | | |
| --- | --- | --- | --- |
|  | **All Respondents**  N = 114 | **CF Physician Experience**  **<15 years**  N = 53 | **CF Physician Experience**  **≥15 years**  N = 61 |
|  | n (%) | n (%) | n (%) |
| **Tissue diagnosis of cancer** | 74 (65%) | 31 (59%) | 43 (70%) |
| **CF-related ESRD requiring dialysis** | 50 (44%) | 18 (34%) | 32 (52%) |
| **Inadequate nutritional status (i.e. BMI <18)** | 33 (29%) | 16 (30%) | 17 (28%) |
| **Other (free text response)** | 31 (27%) | 15 (28%) | 16 (26%) |
| **CF-related liver cirrhosis** | 18 (16%) | 7 (13%) | 11 (18%) |
| **CF-related diabetes, poorly controlled** | 18 (16%) | 9 (17%) | 9 (15%) |
| **Depression, anxiety** | 10 (9%) | 4 (8%) | 6 (10%) |
| **Pulmonary hypertension** | 1 (1%) | 1 (2%) | 0 |
| **Osteoporosis** | 1 (1%) | 0 | 1 (2%) |
| **CF-related sinus disease, extensive** | 0 | 0 | 0 |
| **Gastro-esophageal reflux disease** | 0 | 0 | 0 |
| **Skipped question^ⱡ^** | 12 (11%) | 8 (15%) | 4 (7%) |
| **^ⱡ^** Assumption: none of these would prevent referral for lung transplant evaluation  CF = cystic fibrosis, ESRD = end-stage renal disease, BMI = body mass index | | | |
